# Primary Splenic Diffuse Large B-cell Lymphoma: An Atypical Presentation

**DOI:** 10.7759/cureus.40793

**Published:** 2023-06-22

**Authors:** Sarmad Pirzada, Amana Hasnain, Ali Abbas Mankani, Ibrahim Zahid

**Affiliations:** 1 Internal Medicine, Trinity Health Livonia, Livonia, USA; 2 Internal Medicine, Dow University of Health Sciences, Civil Hospital Karachi, Karachi, PAK; 3 Internal Medicine, Western Michigan University Homer Stryker M.D. School of Medicine, Kalamazoo, USA

**Keywords:** diffuse large b-cell lymphoma, gi malignancy, spleen, splenectomy, primary splenic dlbcl

## Abstract

Primary splenic diffuse large B-cell lymphoma (PS-DLBCL) is an extremely rare type of non-Hodgkin's lymphoma. It typically presents with abdominal pain and a rapidly enlarging mass, often accompanied by B symptoms. Here, we present a rare presentation of PS-DLBCL in a 54-year-old woman who experienced splenomegaly. A CT scan of her abdomen revealed an enlarged spleen measuring 12 x 15 x 14 cm with a hypodense lesion. Confirmation of diffuse large B-cell lymphoma was obtained through a splenic core biopsy. A subsequent positron emission tomography scan showed a large hypermetabolic and centrally necrotic infiltrative splenic mass without any evidence of pathology in other parts of the body. The patient's condition was classified as stage I PS-DLBCL, and she underwent treatment with R-CHOP (rituximab, cyclophosphamide, doxorubicin, vincristine, and prednisone) chemotherapy for four cycles. This case report highlights the unique presentation of diffuse large B-cell lymphoma with exclusive involvement of the spleen and discusses the potential therapeutic role of radiation therapy and R-CHOP without the need for splenectomy.

## Introduction

Although the spleen is frequently involved in patients with non-Hodgkin's lymphoma (NHL), primary splenic diffuse large B-cell lymphoma (PS-DLBCL) without the involvement of any other organ is an extremely rare anatomical subtype of NHL, with an incidence rate of less than 1% of NHLs and less than 2% of all lymphomas [1,2]. While many different criteria have been used to define this primary neoplasm of the spleen, the most appropriate way to define this disorder would be an NHL that is confined to the spleen or involves splenic hilar lymph nodes [3].

PS-DLBCL most commonly presents in older men with abdominal pain, splenomegaly, splenic masses, high lactate dehydrogenase (LDH) levels, and B symptoms such as fever, night sweats, and unexplained weight loss [4]. However, 40% of cases of PS-DLBCL have been reported to present without B symptoms as well. In addition to neoplastic disorders such as diffuse large B-cell lymphoma (DLBCL) and myeloproliferative disorders, more common causes of splenomegaly include hemolytic disorders, liver disease, collagen storage diseases, and acute or chronic infections such as malaria, syphilis, infectious mononucleosis, HIV, and tuberculosis [5,6].

Due to the rare incidence of this lymphoma, there is a scarcity of data available on the ideal diagnostic and management strategies. Splenectomy used to be considered an important intervention for the diagnosis of PS-DLBCL. However, the recent emergence of less invasive splenic biopsies and radiologic investigations has alleviated the need for splenectomy [7]. In this case, we describe a case of PS-DLBCL with atypical age, gender, and presentation and highlight our diagnostic and therapeutic approaches.

## Case presentation

A 54-year-old female presented to the emergency department complaining of left-sided abdominal fullness persisting for two weeks. The patient reported experiencing ongoing abdominal discomfort, occasionally radiating to her shoulders. She denied any associated symptoms such as fever, unintentional weight loss, or night sweats. Her family history was significant for colon cancer in her mother. Physical examination revealed a non-palpable spleen and a non-tender abdomen in the left upper and middle quadrants with no distention, lymphadenopathy, or organomegaly. Complete blood count with differential and peripheral smears were unremarkable. Our patient also had an elevated LDH level of 430 IU/L (Table 1).

**Table 1:** Initial laboratory results upon presentation.

Lab	Results	Reference
Hemoglobin	11.7	11.1-14.5 g/dL
Hematocrit	36.4%	35.4-42.0%
RBC count	4.09 × 10^6/microL	3.9-5.5 × 10^6/microL
Mean corpuscular volume	83.1 fL	76.0-96.0 fL
Mean corpuscular hemoglobin	28.1 pg	26.0-32.0 pg
Mean corpuscular hemoglobin concentration	31.6 g/dL	32.0-36.0 g/dL
WBC count	12.0 × 10^9/L	4.0-10.0 × 10^9/L
Neutrophils	70.1%	40-75%
Lymphocytes	20.4%	20-45%
Eosinophils	1.5%	1-6%
Monocytes	7.6%	2-10%
Basophils	0.4%	0-1%
Platelets	250 × 10^9/L	150-400 × 10^9/L
Na+	138 mmol/L	136-145 mmol/L
K+	4.4 mmol/L	3.5-5.0 mmol/L
Cl-	98 mmol/L	95-106 mmol/L
Glucose	90 mg/dL	65-99.0 mg/dL
Ca2+	10.2 mg/dL	8.7-10.2 mg/dL
Blood urea nitrogen	13 mg/dL	6-24 mg/dL
Creatinine	0.74 mg/dL	0.76-1.27 mg/dL
Alkaline phosphatase	180 UI/L	0-130 UI/L
Alanine aminotransferase	50 UI/L	0-40 UI/L
Aspartate aminotransferase	50 UI/L	0-40 UI/L
Total bilirubin	0.3 mg/dL	0.0-1.2 mg/dL
Albumin	2.2 g/dL	3.5-5.5 g/dL
Total protein	6.3 g/dL	6.0-8.5 g/dL
Serum lactate dehydrogenase	407 U/L	140-271 U/L
C-reactive protein	6.0 mg/dL	0.0-0.5 mg/dL
Prothrombin time	13.3 sec	9.4-12.5 sec
Cardiolipin IgM	19.3 MPL	<12.5 MPL

**Figure 1:**
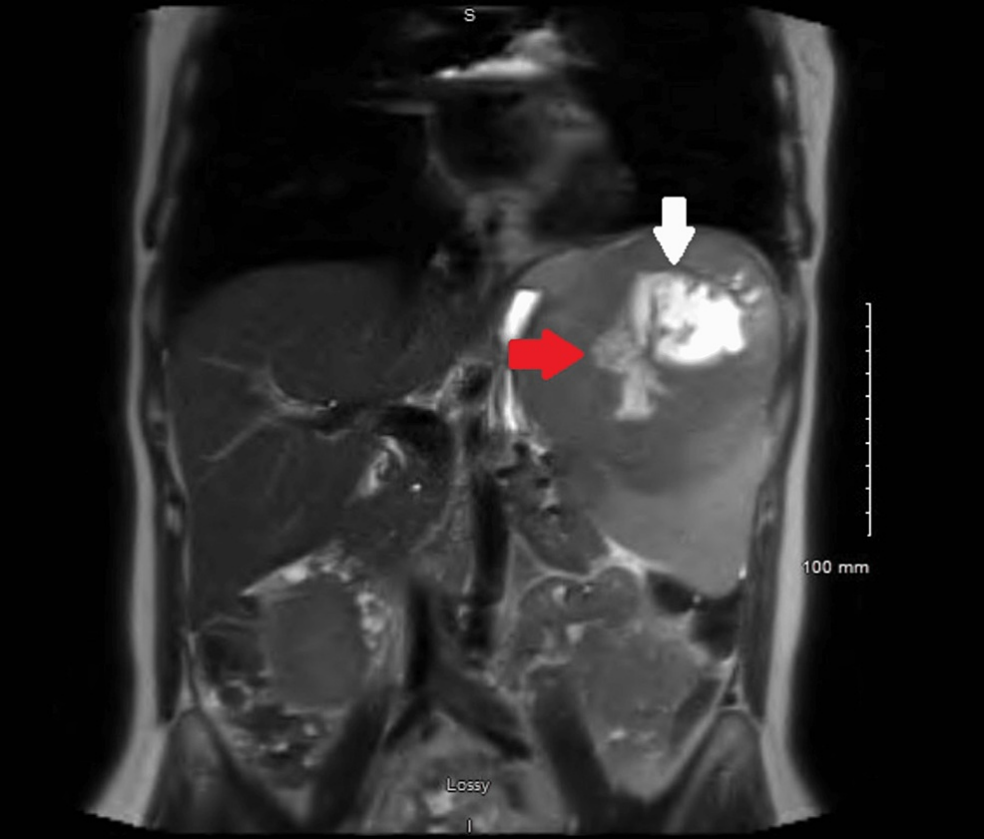
MRI of the abdomen. White arrow: Large aggressive-appearing splenic mass (13 cm). Red arrow: Mass invading the splenic vein for a distance of 5 cm.

CT scan of the abdomen showed an enlarged spleen of 12 x 15 x 14 cm with a hypodense lesion. Magnetic resonance imaging showed a large 13 cm splenic mass with a sizable region of internal necrosis with neoplastic invasion of the splenic vein for a distance of 6 cm, concerning for a splenic angiosarcoma (Figure 1). There was also probable wall invasion of the involved splenic vein; the involved portion of the splenic vein was substantially expanded, and the tumor thrombus within it was completely occlusive. This mass also probably invaded an intrasplenic vein inferiorly.

A positron emission tomography (PET) scan exhibited a large hypermetabolic and centrally necrotic infiltrative splenic mass with no evidence of disease in other sites. Interventional radiology performed a splenic core biopsy, and the pathology showed DLBCL, germinal center B-cell subtype. This was classified as stage I PS-DLBCL. Fluorescence in situ hybridization (FISH) analysis ruled out double/triple hits. A treatment plan was formulated to use R-CHOP (rituximab, cyclophosphamide, doxorubicin, vincristine, and prednisone) therapy for four cycles, along with radiation, to treat the bulky stage I disease. The patient is responding well to the initial cycles of treatment.

## Discussion

While secondary involvement of the spleen is common in lymphomas, primary involvement of the spleen is a very rare finding [8]. Histopathologically, primary splenic lymphomas (PSL) can be divided into indolent subtypes such as splenic marginal zone lymphoma and follicular lymphoma, or more aggressive subtypes such as hepatosplenic T-cell lymphoma or DLBCL, which is the most common subtype, as seen in this case [9]. With a male-to-female incidence ratio of 1.35:1, PS-DLBCL is more commonly found in older males but may occur at any age [5]. In fact, the incidence of DLBCL is only 5.8 per 100,000 persons [10].

PSL may also be associated with the hepatitis C virus (HCV) and human immunodeficiency virus (HIV). These infections may contribute to the development of lymphomas through several different mechanisms. In fact, a strong HCV seroprevalence has been documented in patients with early-stage DLBCL, with almost a 100% co-occurrence. Positive HIV or HCV infection may affect lymphocytes directly or cause chronic antigen stimulation, which can lead to lymphocyte proliferation. Additionally, the HCV-encoded E2 protein may cause the CD81 protein to activate B-cells, reducing cell-mediated apoptosis and increasing double DNA strand breaks [11,12]. Further research is still needed to fully understand the role of HCV and HIV infection in causing lymphomas. However, our patient tested negative for both HIV and HCV.

PS-DLBCL is a rare entity, with an incidence of less than 1%. Symptoms may include splenomegaly, general weakness, and left upper quadrant pain [13]. Approximately one in three people with DLBCL also experience fevers, night sweats, and unexplained weight loss [14]. These are referred to as "B symptoms." In our case, there were no B symptoms, severe abdominal pain, or weakness.

The first sonographic sign of DLBCL is frequently a hypoechoic lesion on ultrasound. However, different subtypes of splenic lymphomas cannot be differentiated sonographically. On CT scans, you may see a well-circumscribed hypodense nodular mass within the spleen [15].

Commonly seen laboratory anomalies include cytopenias, elevated levels of B2 globulin and erythrocyte sedimentation rate, and anemia [15]. In this instance, we encountered a 54-year-old female patient with no notable medical background, whose laboratory tests displayed increased levels of LDH and C-reactive protein. Her main complaint at presentation was abdominal fullness and she did not report any B symptoms.

A definitive diagnosis is made by means of a splenectomy or a core needle splenic biopsy. Unlike other reported cases like those reported by Safe et al. [9] and Alsamman et al. [16], our case presented with significant proximity to the abdominal wall, making it accessible to interventional radiology (IR)-guided biopsy for diagnosis, eliminating the need for splenectomy.

Treatment options for PS-DLBCL include splenectomy (which serves as both a diagnostic and treatment modality), local radiation therapy, chemotherapy, or a combination of these modalities [17]. Unfortunately, there is no consensus regarding the best treatment option for PS-DLBCL. However, the inclusion of rituximab in standard CHOP chemotherapy has drastically shown to improve response and survival rates in DLBCL patients [18]. Since the patient in our case had a good functional status, it made her a good candidate for R-CHOP therapy with radiation.

Although rare, a few cases of PSL have previously been reported in the literature as well. Kattepur et al. reported a case of a 50-year-old female patient who presented with a history of abdominal pain for two months. A CT scan showed a large solid mass arising from the lower pole of the spleen measuring 7.5 × 6 cm. Histopathological examination of the spleen led to the diagnosis of PSL [19]. Similarly, Sachin B Ingle and Chitra R Hinge Ingle reported a case of PS-DLBCL in a 41-year-old female presenting with abdominal pain and weight loss, massive splenomegaly, and hypersplenism, without fever and lymphadenopathy [20]. Other than experiencing no weight loss, our patient shared similar symptoms with the 41-year-old patient.

Alsamman et al. also reported a case highlighting the potential complications of splenic vein occlusion by lymphoma cells in a 64-year-old male with DLBCL [16]. The case described the consequence of splenic vein occlusion, causing left-sided portal hypertension and eventually leading to isolated gastric varices in the patient. In our case, there were no complications associated with spleen involvement.

Our case highlights a unique presentation of DLBCL, where the patient only experienced occasional abdominal discomfort radiating to her left shoulder, while all other symptoms were non-significant. Her CT scan revealed isolated splenomegaly. This shows that primary splenic DLBCL is a rare but extremely important differential diagnosis for physicians to consider when they encounter a patient with isolated splenomegaly. Considering the uniqueness of this presentation, it brings into question what we currently know about PS-DLBCL. Due to the rare incidence of this disease, there is a lack of prior studies on this disorder, which we believe warrants further research on this topic.

## Conclusions

There is still much that we do not know about the appropriate techniques for managing and diagnosing PS-DLBCL. Our case involves a 54-year-old female with PS-DLBCL, who was diagnosed through an IR-guided splenic core biopsy and treated with four cycles of R-CHOP paired with radiation therapy. This case highlights the significance of considering primary splenic DLBCL, albeit rare, in the differential diagnosis when a patient presents with abdominal fullness, splenomegaly, and non-specific clinical findings, and emphasizes the importance of chemotherapy as a treatment option. When PSL-DLBCL is diagnosed in its early stages, there is a higher chance of a better prognosis and more effective management. We hope that the presentation of this case report, which discusses a case of PS-DLBCL with isolated splenomegaly, adds value to the current literature on DLBCL and contributes to the understanding of this condition.
